# PET imaging of CXCR4 expression using [^18^F]AlF-NOTA-QHY-04 for hematologic malignancy and solid tumors

**DOI:** 10.7150/thno.99025

**Published:** 2024-09-30

**Authors:** Kai Cheng, Shijie Wang, Tianxin Liu, Jinli Pei, Shasha Wang, Jingru Liu, Kunlong Zhao, Yuxi Luo, Shengnan Xu, Jinming Yu, Jie Liu

**Affiliations:** 1 Shandong Provincial Key Laboratory of Precision Oncology, Shandong Cancer Hospital and Institute, Shandong First Medical University and Shandong Academy of Medical Sciences, Jinan, China.; 2 Department of PET/CT Center, Shandong Cancer Hospital and Institute, Shandong First Medical University and Shandong Academy of Medical Sciences, Jinan, China.; 3 Lung Cancer Center, West China Hospital, Sichuan University, Chengdu, China.; 4 Department of Radiation Oncology, Shandong Cancer Hospital and Institute, Shandong First Medical University and Shandong Academy of Medical Sciences, Jinan, China.; **First Authors:** Kai Cheng, Shijie Wang and Tianxin Liu contributed equally to this work.

**Keywords:** CXCR4, PET/CT, solid tumors, lymphoma, SCLC

## Abstract

C-X-C motif chemokine receptor 4 (CXCR4) is an attractive target for the diagnosis and treatment of cancers. Here, we aimed to develop a new CXCR4-targeted PET tracer, and to investigate the translational potential for noninvasive imaging of CXCR4 expression in various cancer entities through preclinical and pilot clinical studies.

**Methods** [^18^F]AlF-NOTA-QHY-04 was synthesized and evaluated by cellular uptake, blocking and biolayer interferometry studies *in vitro*. The pharmacokinetics, biodistribution, and imaging specificity were researched in tumor-bearing mice. [^18^F]AlF-NOTA-QHY-04 PET/CT imaging was performed on 55 patients with different types of cancers. Correlations between *ex vivo* CXCR4 expression and PET parameters, and CXCR4 expression characteristics in different tumors were analyzed by histopathological staining in patients.

**Results** [^18^F]AlF-NOTA-QHY-04 was prepared with high radiolabeling yield and radiochemical purity, exhibiting good stability, high binding affinity and specificity for CXCR4*.* NCI-H69 (small cell lung cancer, SCLC) tumor-bearing mice showed the highest tumor uptake (4.98 ± 0.98%ID/mL, *P* < 0.0001) on PET imaging except for Daudi lymphoma xenograft model, which was consistent with the results of cellular and histological analyses. Patients with diffuse large B-cell lymphoma showed the highest tumor uptake (SUV_max_, 11.10 ± 4.79) followed by SCLC patients (SUV_max_, 7.51 ± 3.01), which were both significantly higher than other solid tumors (*P* < 0.05). The radiotracer uptake of high-grade gliomas is significantly higher than that of low-grade gliomas (3.13 ± 0.58 vs. 1.18 ± 0.51, *P* = 0.005). Significant higher tumor-to-normal brain ratio of [^18^F]AlF-NOTA-QHY-04 than [^18^F]FDG was found in primary brain tumors (62.55 ± 43.24 vs 1.70 ± 0.25, *P* = 0.027). Positive correlations between *ex vivo* CXCR4 expression and [^18^F]AlF-NOTA-QHY-04 uptake (all *P* < 0.01) were recorded. Multicolor immunofluorescence staining indicated the high tracer uptake in certain patients was mainly due to the high expression of CXCR4 in tumor cells, followed by macrophages.

**Conclusion** The CXCR4-targeted radiotracer [^18^F]AlF-NOTA-QHY-04 was successfully prepared with favorable yield, high specificity and binding affinity to CXCR4. Preclinical and pilot clinical studies demonstrated its feasibility and potential application in precise diagnosis for not only lymphoma but also SCLC and glioma. [^18^F]AlF-NOTA-QHY-04 PET/CT can also provide a complementary mapping for brain tumors to [^18^F]FDG PET/CT.

## Introduction

CXCR4 is a typical seven transmembrane G protein-coupled receptor highly expressed by various human cancers and crucially involved in tumor growth, invasion, angiogenesis, and metastasis, rendering this receptor as an attractive target for cancer diagnosis, treatment, and prognosis 1-4. As such, CXCR4-directed PET agents that can noninvasive *in vivo* quantification of CXCR4 expression display significant clinical value not only for guiding precise diagnosis and prognosis of several cancers but also for the patient selection suitable for CXCR4-targeted therapy 5,6, such as pharmacologic or endoradiotherapeutic interventions 7,8.

Currently, various CXCR4-targeted PET agents have been developed by radiolabeling CXCR4 inhibitors or antagonists 9. Among them, ^68^Ga-Pentixafor, a ^68^Ga-labeled cyclic pentapeptide 10, has been most studied in a wide variety of clinical settings in oncology, particularly in hematological malignancies 6,11,12. Although those studies have demonstrated that ^68^Ga-pentixafor exhibits not only high affinity and selectivity for CXCR4 but also rapid renal excretion, and low background accumulation, the superior radionuclide properties of ^18^F with a longer half-life (109.8 minutes) and greater spatial imaging resolution than ^68^Ga 13,14, highly motivate the development of ^18^F-labeled CXCR4 PET agents. However, the clinical application of the reported ^18^F-labeled CXCR4-targeted agents, such as Al[^18^F]NOTA-T140 15, [^18^F]AlF-NOTA-pentixather 16, [^18^F]MCFB 17, [^18^F]RPS-544 18, has been hindered by the lowered imaging contrast due to their high retention in blood, peripheral tissues, intestines or liver and low uptake in CXCR4-expressing tumors. Hence, there is an urgent need to develop a new ^18^F-labeled CXCR4-targeted radiotracer endowing with high imaging contrast and CXCR4-specific accumulation both in solid tumors and hematologic neoplasms.

Recently, we developed a new ^18^F-labeled CXCR4-targeting tracer ([^18^F]AlF-NOTA-QHY-04) based on a new cyclic peptide with high serum stability and high CXCR4 affinity 19, and successfully applied this tracer in the early and noninvasive detection of radiation-induced lung injury in rat models and patients 20. Encouraged by the safety and feasibility of [^18^F]AlF-NOTA-QHY-04 in monitoring radiation-induced lung injury, this study comprehensively investigated its binding affinity, specificity and imaging efficacy *in vitro*, in mouse tumor-bearing models and *ex vivo*, and evaluated the applicability of this ^18^F-labled tracer for specific imaging of CXCR4 expression in 55 patients with 8 different types of cancers including solid and hematologic neoplasms. We also compared the performance of [^18^F]AlF-NOTA-QHY-04 PET/CT and ^18^F-FDG PET/CT in the diagnosis of patients with brain tumors. Moreover, we conducted a correlation analysis between radiotracer uptake in 16 SCLC patients and immunohistochemical staining of CXCR4 expression, and further analyzed the CXCR4 expression characteristics in different patients by mIF staining.

## Materials and methods

### Chemistry and radiolabeling

The precursor NOTA-QHY-04 was acquired from Nanchang Tanzhen Bio Co., Ltd. (Nanchang, China) with a high purity (> 95%) and other commercial reagents and solvents were used without further purification. NOTA-QHY-04 was radiolabeled with [^18^F]F^-^ as we reported previously 20. The synthesis procedure of [^18^F]AlF-NOTA-QHY-04 is delineated in the supplemental materials. A radio-high-performance liquid chromatography (radio-HPLC) (1525 Series, Waters, USA) was utilized to examine the radiochemical purity (RCP).

### Stability and biolayer interferometry (BLI) binding assays

The *in vitro* and *in vivo* stability of [^18^F]AlF-NOTA-QHY-04 were evaluated by radio-HPLC to examine the RCP after different incubation periods. The binding affinity of the unlabeled precursor to CXCR4 was measured using the biolayer interferometry Octet Red96 system (PALL ForteBio, USA). The details are supplied in the supplemental materials.

### Cell culture and tumor-bearing models

Six human tumor cell lines (A549, Daudi, NCI-H69, U251, MDA-MB-231 and MIA PaCa-2) and CXCR4-overexpressed A549 cells (A549/CXCR4) were used in this study. Detailed descriptions for cell culture and tumor transplantation are provided in the supplemental materials. All studies involving animals were performed in accordance with the guidelines of the Animal Care and Use Committee of Shandong Cancer Hospital and Institute, the Shandong First Medical University (Jinan, China).

### Flow cytometry, immunofluorescence staining and cell uptake assays

CXCR4 expression levels in different tumor cells were analyzed by flow cytometry and immunofluorescence staining, which were performed as described in the supplemental materials. For the uptake study, cells (4×10^5^) were incubated with [^18^F]AlF-NOTA-QHY-04 (37 kBq/well), with or without NOTA-QHY-04 precursor or AMD3100 (a specific CXCR4 antagonist) (2 μg/well) as a blocking agent for 60 min (*n* = 3). Then cells were washed with PBS and collected for radioactivity measurement with a γ-counter (2470 Wizard2, PerkinElmer). Cellular uptake of [^18^F]AlF-NOTA-QHY-04 was indicated as the percentage of added radioactive dose.

### Small-animal PET imaging and *ex vivo* biodistribution studies

The xenografted mice were injected with [^18^F]AlF-NOTA-QHY-04 (3.70 ± 0.74 MBq) in about 150 μL saline via the tail vein. All the PET scans were performed with micro-PET/CT (IRIS PET/CT, Inviscan, France) and the radioactivity were expressed as %ID/mL. Detailed procedures for dynamic, static imaging and blocking studies are provided in the supplemental materials. Biodistribution studies were conducted using mice with xenografted A549/CXCR4 tumors with and without blocking (*n* = 3). The mice were sacrificed immediately after static PET/CT 1 h post injection (p.i.), then the tumors and organs were quickly collected, weighed and counted for radioactivity by a γ-counter. The γ-counter activity readings were background- and decay-corrected and represented as the percentage of injected dose per gram of tissue (%ID/g).

### PET/CT imaging in cancer patients

Potential eligible patients with newly diagnosed tumor were recruited from Shandong Cancer Hospital and institute from November 2022 to December 2023. A total of 55 patients including 8 tumor types were represented in this study (diffuse large B-cell lymphoma (DLBCL), *n* = 6; SCLC, *n* = 30; non-small cell lung cancer (NSCLC), *n* = 4; triple-negative breast cancer (TNBC), *n* = 3; pancreatic carcinoma, *n* = 3; glioma, *n* = 7; hepatocellular carcinoma (HCC), *n* = 1; and colorectal carcinoma (CRC), *n* = 1). The [^18^F]AlF-NOTA-QHY-04 imaging study in patients was approved by the Institutional Review Board of Shandong Cancer Hospital and Institute (no. SDZLEC2023-256-02), and the patients provided their informed consent before inclusion in this study. In this prospective observational study, [^18^F]AlF-NOTA-QHY-04 PET/CT scans should not affect the normal process of routine examinations and treatment as usual. Patients were enrolled according to the following criteria: (1) newly diagnosed cancer patients with visible tumors on conventional imaging; (2) histopathologically confirmed diagnosis; (3) age ≥ 18 years; (4) Eastern Cooperative Oncology Group (ECOG) performance status score of 0-2; (5) no prior cancer-directed therapy; (6) ability to undergo [^18^F]AlF-NOTA-QHY-04 PET/CT scanning before treatment initiation. Specifically, regarding histological confirmation, we followed the standard clinical practice guidelines for each type of cancer 21-26, and provided detailed descriptions of our approach for obtaining histological evidence in the supplemental materials. All patients included in this study were treatment-naïve at the time of [^18^F]AlF-NOTA-QHY-04 PET/CT imaging, to ensure that CXCR4 expression levels were not altered by treatments. PET/CT scans were performed within one week of pathological confirmation and carefully coordinated with the patient's clinical teams to avoid any delays in the initiation of treatment. Seven patients from them also underwent [^18^F]FDG PET/CT scan. These patients fasted for at least 6 hours before [^18^F]FDG PET/CT imaging. The methods for PET/CT scanning and image analysis were the same as previously reported 20, and detailed in the supplemental materials.

### Immunohistochemical (IHC) and multicolor immunofluorescence (mIF) staining studies

IHC staining was performed to identify the expression of CXCR4 in tumor tissues from xenograft models and patients. Specifically, to clarify if [^18^F]AlF-NOTA-QHY-04 PET imaging can represent the expression of CXCR4 in tumor, we analyze the correlation between tumor uptake and CXCR4 expression in 16 SCLC patients. For IHC, the anti-CXCR4 rabbit monoclonal antibody (1:200, ab124824, Abcam) was used. For mIF staining, the sequence of primary antibodies and Opal fluorophores for patient samples was anti-panCK/Opal 480, anti-CD3/Opal 570, anti-NE/Opal 520, anti-CXCR4/Opal 620, anti-CD20/Opal 690, and anti-CD68/Opal 780.

### Statistical analysis

GraphPad Prism (version 8.3.0) was utilized for the statistical analysis, and the descriptive results were displayed as mean ± standard deviation (SD). Differences between two groups and multiple groups were analyzed by unpaired Student's *t*-test and One-way ANOVA (with Tukey's multiple comparisons test), respectively. Differences with a *P* value < 0.05 shown with one asterisk (*) were determined as statistically significant.

## Results

### Synthesis and characterization of [^18^F]AlF-NOTA-QHY-04

**Figure 1:**
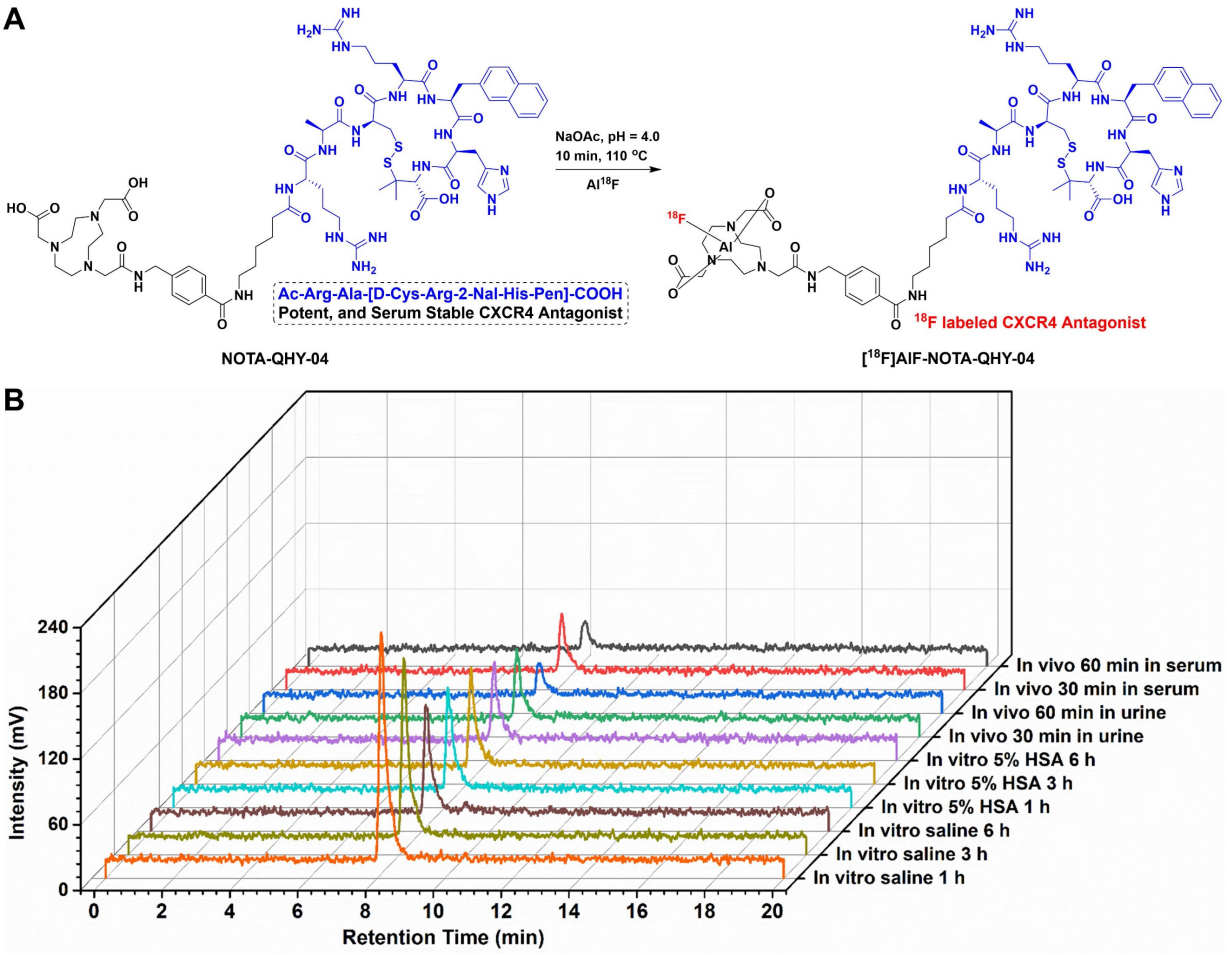
Synthesis and characterization of [^18^F]AlF-NOTA-QHY-04. (A) Radiosynthesis of [^18^F]AlF-NOTA-QHY-04. (B) Radio-HPLC analysis of the tracer stabilities under different conditions.

The precursor NOTA-QHY-04 was successfully prepared according to the synthesis procedure summarized in Figure S1 and identified through the use of mass spectrometry (Figure S2) and ^1^H NMR spectrum (Figure S3). As shown in Figure 1A, a potent CXCR4 antagonist (Ac-Arg-Ala-[D-Cys-Arg-2-Nal-His-Pen]-COOH) was selected as the CXCR4 ligand due to the high affinity and selectivity against CXCR4 19. The precursor was efficiently radiolabeled via the Al^18^F labeling method with a non-decay corrected radiolabeling yield of 59 ± 10% (*n* = 22) and the specific activity was calculated as 30 ± 3 GBq/μmol at the end of the synthesis (nearly 30 min). The RCP of [^18^F]AlF-NOTA-QHY-04 was more than 98% after purification as determined by radio-HPLC. The logD value of [^18^F]AlF-NOTA-QHY-04 was -2.21 ± 0.06, indicating [^18^F]AlF-NOTA-QHY-04 was highly hydrophilic. The detailed results of quality control are supplied in Table S1.

The *in vitro* and *in vivo* stability of [^18^F]AlF-NOTA-QHY-04 were assessed by radio-HPLC. The radiochemical purity of [^18^F]AlF-NOTA-QHY-04 remained above 95% after incubation in saline or 5% human serum albumin solution even for 6 h (Figure 1B). Furthermore, the RCP of the radiotracer was higher than 90% in the blood and urine of BALB/c nude mice until 60 min p.i. These results suggested [^18^F]AlF-NOTA-QHY-04 has good stability both *in vitro* and *in vivo*.

### Specific binding of [^18^F]AlF-NOTA-QHY-04 to CXCR4 *in vitro*

**Figure 2:**
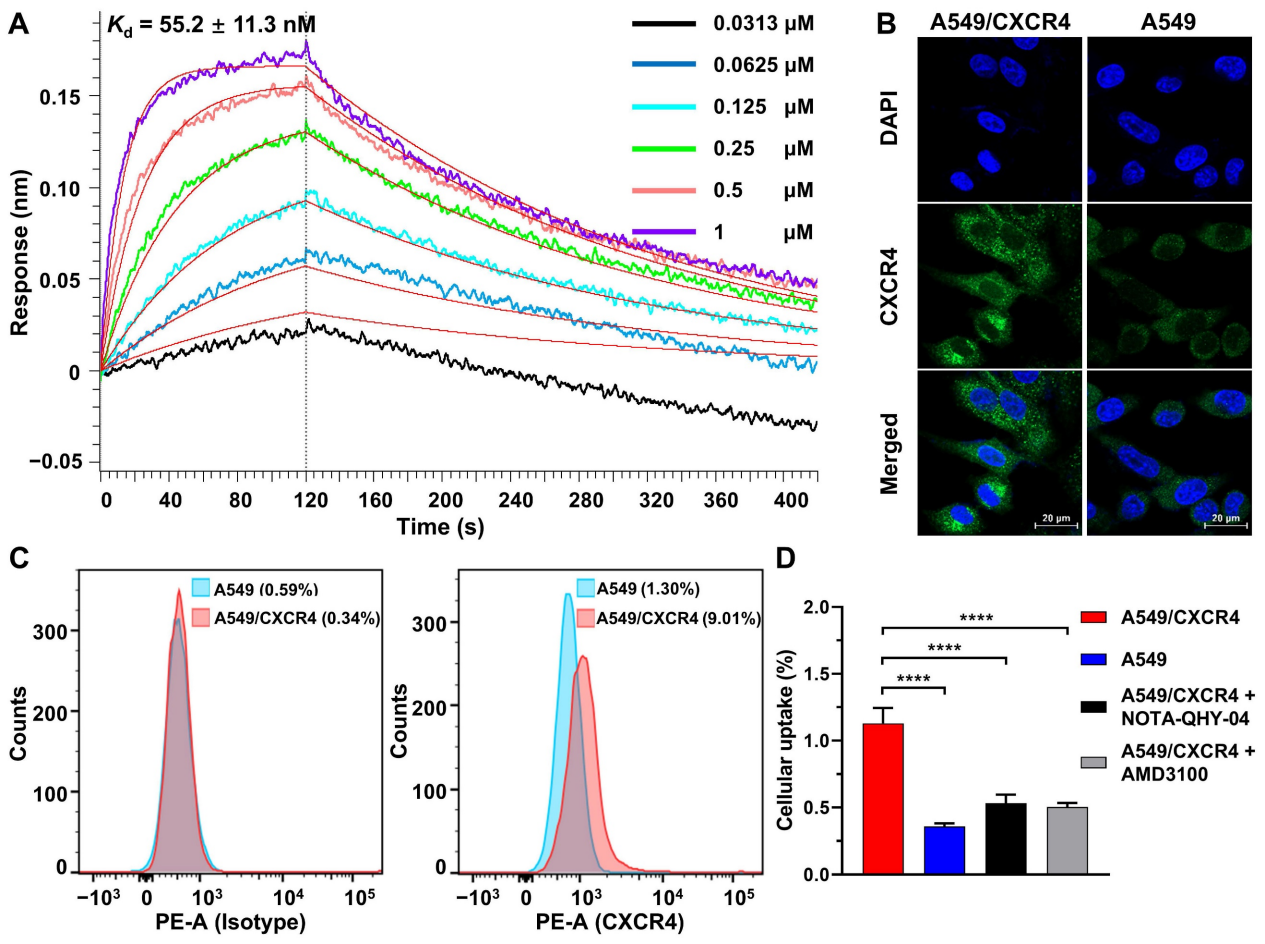
Binding affinity and cellular uptake. (A) Determination of the binding affinity between CXCR4 and NOTA-QHY-04 by biolayer interferometry. (B) Representative IF staining of A549 and A549/CXCR4 cells with the anti-CXCR4 antibody and DAPI. Scale bar displayed as 20 μm. (C) Flow cytometry histograms of A549/CXCR4 and A549 cells incubated with PE-conjugated anti-human CD184 (CXCR4) antibody along with the isotype control antibody. (D) [^18^F]AlF-NOTA-QHY-04 uptake in A549/CXCR4 and A549 cells with or without blocking at 60 min (*n* = 3). ****P < 0.0001.

The binding affinity of NOTA-QHY-04 for CXCR4 was measured using BLI. The *K*_d_ values for NOTA-QHY-04 and [^nat^F]AlF-NOTA-QHY-04 were determined to be 55.2 ± 11.3 nM (Figure 2A) and 41.3 ± 5.5 nM (Figure S4), respectively, indicating the high affinity of the radiotracer towards CXCR4. CXCR4-transfected A549 cell line (A549/CXCR4), confirmed to have higher CXCR4 expression at both the mRNA and protein levels than the wild type through immunofluorescence, flow cytometry, western blot and qRT-PCR (Figure 2B and 2C, Figure S5), was used as a CXCR4-positive cell line for cell uptake assays. As shown in Figure 2D, the uptake of [^18^F]AlF-NOTA-QHY-04 was significantly higher in A549/CXCR4 cells than in A549 cells, and the uptake could be blocked by the addition of excess unlabeled precursor or AMD3100. Therefore, [^18^F]AlF-NOTA-QHY-04 holds high affinity and specificity to CXCR4 *in vitro*.

### Micro-PET/CT for Specific binding studies and biodistribution studies

**Figure 3:**
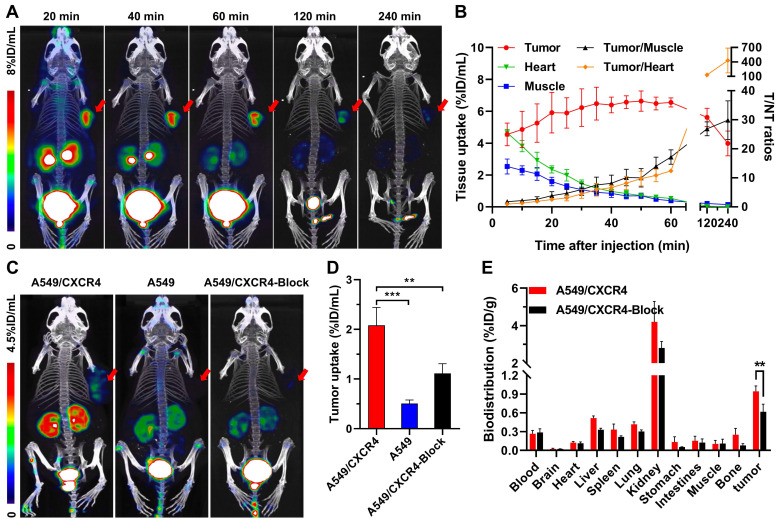
Representative dynamic micro-PET images, time-activity curves and biodistribution of [^18^F]AlF-NOTA-QHY-04. (A) Representative dynamic PET images of NCI-H69 xenograft mice at different time p.i. of [^18^F]AlF-NOTA-QHY-04. (B) Dynamic time-activity curves in muscle, heart and tumor tissues and the tumor-to-normal tissue (T/NT) ratios for 4 h p.i. (*n* = 4). (C) Representative PET images and (D) tumor uptake of [^18^F]AlF-NOTA-QHY-04 in A549, A549/CXCR4 and blocked A549/CXCR4 xenograft mice at 1 h p.i. (*n* = 3). (E) Biodistribution of [^18^F]AlF-NOTA-QHY-04 in A549/CXCR4-bearing mice about 1 h p.i. (*n* = 3). The tumors are delineated in red arrows. **P < 0.01, ***P < 0.001.

Based on our previous work that [^18^F]AlF-NOTA-QHY-04 shows no acute radiotoxicity 20, so as to confirm the *in vivo* specificity and imaging efficacy, 60-min dynamic PET was firstly performed on mice bearing NCI-H69 subcutaneous tumor xenografts, which showed high CXCR4 expression in the tested wild-type cells (see below). The PET images and time-activity curves showed that the tumor accumulation of [^18^F]AlF-NOTA-QHY-04 was rapid, reaching the highest uptake at about 50 min p.i. and then gradually decreased from 60 min to 240 min p.i. (Figure 3A and 3B). In contrast, the blood, heart, liver, and muscle uptake exhibited quick elimination. The tumor-to-muscle ratio and tumor-to-heart ratio increased gradually over the total scan time of 60 min. On the basis of the image contrast data and tumor uptake value, the static PET imaging was performed at 60 min after injection of the tracer. Next, target specificity was further evaluated in A549/CXCR4 and A549 tumor-bearing mice. As expected, the uptake of [^18^F]AlF-NOTA-QHY-04 in A549/CXCR4 xenografts was significantly higher than that in A549 tumors (2.08 ± 0.36 vs. 0.51 ± 0.07%ID/mL, *P* < 0.001, Figure 3C and 3D). Pretreatment with the unlabeled precursor significantly decreased the uptake in A549/CXCR4 tumor (2.08 ± 0.36 vs. 1.11 ± 0.19%ID/mL, *P* < 0.01).

Furthermore, the biodistribution of [^18^F]AlF-NOTA-QHY-04 in A549/CXCR4 tumor-bearing mice was determined by *ex vivo* counting in tissues collected after the above static PET scan. As presented in Figure 3E and Table S2, kidneys showed the highest uptake of [^18^F]AlF-NOTA-QHY-04, while other normal organs showed low radioactivity accumulation, indicating that [^18^F]AlF-NOTA-QHY-04 cleared primarily through the kidneys, which is related to its hydrophilicity. Consistently with the PET findings, high uptake of the tracer was observed in the A549/CXCR4 tumors, and the uptake can be significantly reduced via blocking with an excess of NOTA-QHY-04, which indicated again that the tumor accumulation of [^18^F]AlF-NOTA-QHY-04 was CXCR4 specific.

### Micro-PET/CT for different types of tumors in mice

**Figure 4:**
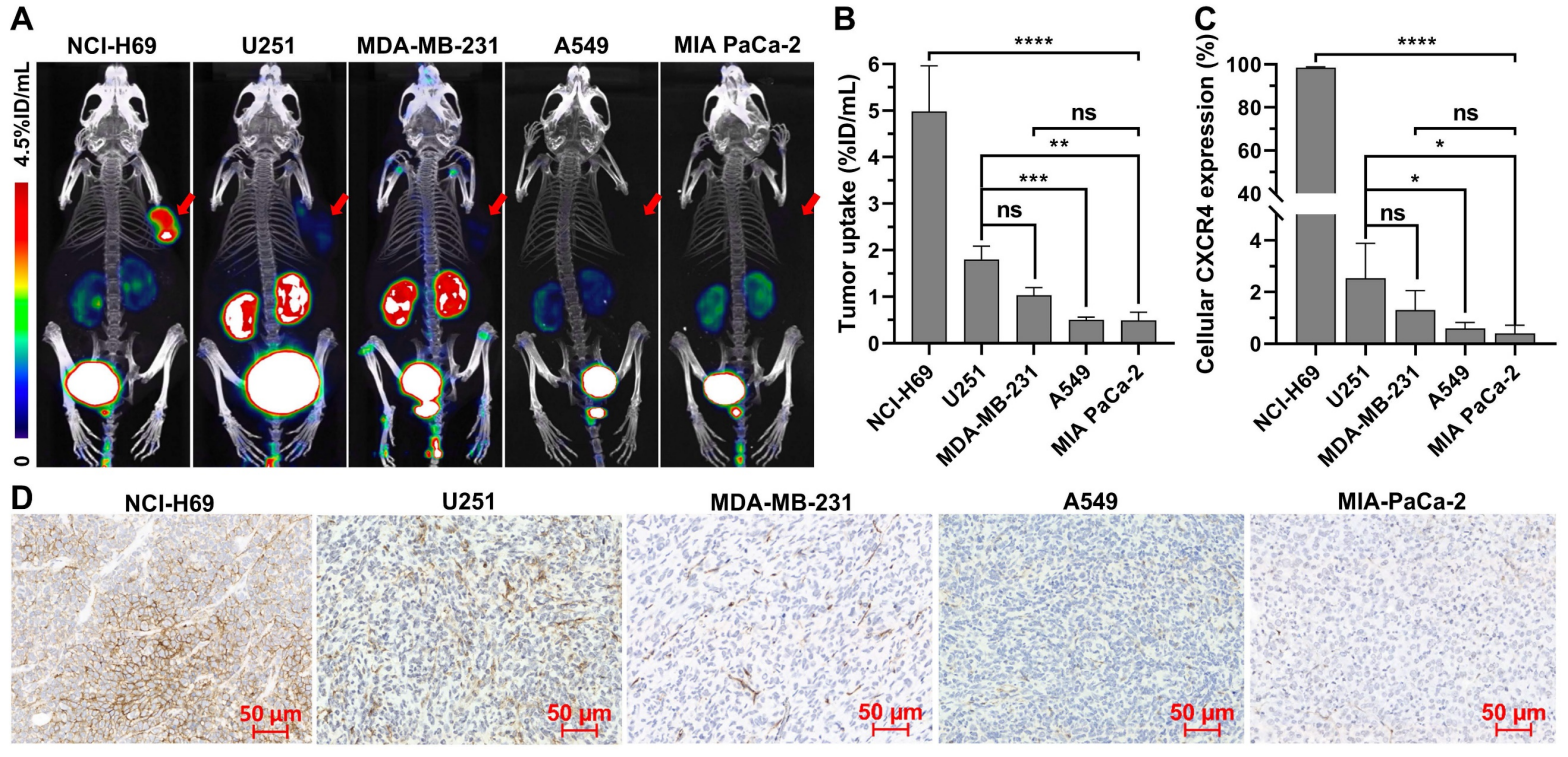
Micro-PET/CT imaging and CXCR4 expression in different types of tumors. (A) Representative static PET images and (B) the uptake of [^18^F]AlF-NOTA-QHY-04 in different types of tumors (*n* ≥ 3). (C) The relative expression of CXCR4 in different types of tumor cells determined by flow cytometry (*n* = 3). (D) CXCR4 immunohistochemistry staining of different tumor xenografts. ns, not statistically significant. *P < 0.05, **P < 0.01, ***P < 0.001, ****P < 0.0001.

To verify whether [^18^F]AlF-NOTA-QHY-04 can be used for evaluation of CXCR4 expression in different types of tumors, PET imaging studies were conducted in multiple types of tumor models, including SCLC (NCI-H69), NSCLC (A549), TNBC (MDA-MB-231), glioma (U251), pancreatic carcinoma (MIA PaCa-2), and lymphoma (Daudi). All the tested tumors were clearly visualized at 1 h after tracer administration except for the A549 and MIA-PaCa-2 tumors (Figure 4A). Quantitative analysis showed that the highest tracer uptake was found in Daudi tumors, which was significantly higher than that in the other solid tumors (Figure S6). Among the solid tumors, NCI-H69 displayed the highest uptake, followed by U251, MDA-MB-231, A549, and MIA PaCa-2 tumor xenografts (Figure 4B). More importantly, these results were in accordance with the cellular and histological analysis of CXCR4 expression by flow cytometry (Figure 4C and Figure S7), immunofluorescence (Figure S8), as well as IHC staining (Figure 4D).

### PET/CT imaging in cancer patients

The detailed information of 55 patients who underwent [^18^F]AlF-NOTA-QHY-04 PET/CT examination was presented in Table 1. The radiotracer was well-tolerated in all patients. No adverse events were recorded in any patients during or after [^18^F]AlF-NOTA-QHY-04 PET/CT imaging. Overall, 54 of 55 patients (98.2%) showed at least one [^18^F]AlF-NOTA-QHY-04-positive lesion. Positive lesions were defined as those with uptake visually higher than the local background, in line with previous studies on CXCR4-targeted PET imaging 27,28. Only one patient with CRC (1.8%) had negative scan.

### PET imaging of patients with different types of tumors

**Figure 5:**
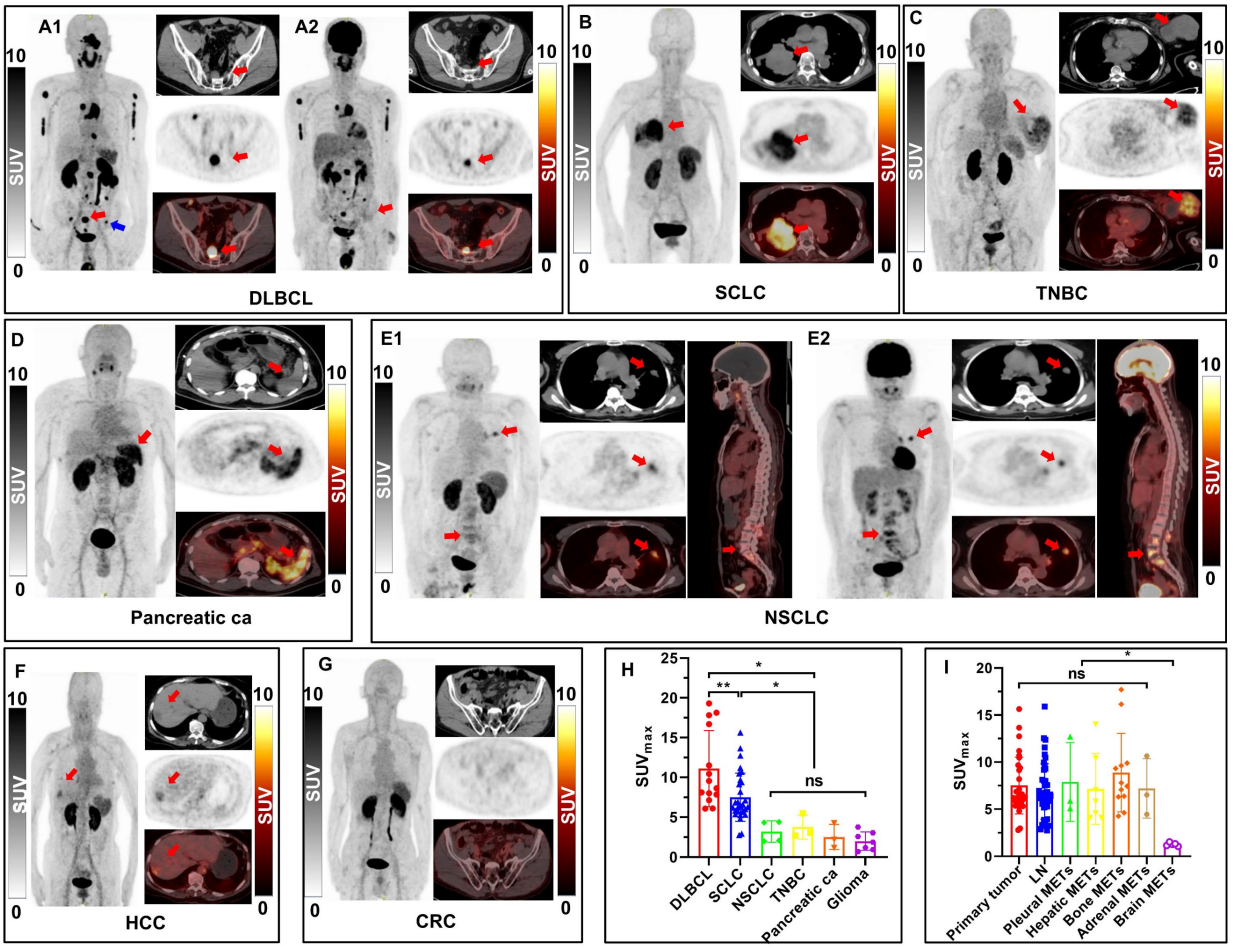
[^18^F]AlF-NOTA-QHY-04 PET/CT scanning of patients with hematologic malignancies or different solid tumors. (A-G) For each case, maximum-intensity projections (MIP) are presented on the left, with corresponding transaxial CT (top), PET (middle), and fused PET/CT (down) images shown on the right. A2 and E2 are [^18^F]FDG PET/CT images, the others are [^18^F]AlF-NOTA-QHY-04 PET/CT images. Red arrows indicate CXCR4-positive tumor lesions; the blue arrow indicates the inguinal lymph node. (H) SUV_max_ of [^18^F]AlF-NOTA-QHY-04 in 6 types of tumor patients. (I) Comparison of SUV_max_ values in primary tumors and metastases of 30 SCLC patients. ns, not statistically significant. *P < 0.05, **P < 0.01.

As illustrated in Figure 5A-G, visual assessment of PET/CT images revealed varying patterns of disease extent and intensity across different types of tumors, including DLBCL, SCLC, NSCLC, TNBC, pancreatic carcinoma, glioma, HCC and CRC. Regarding the quantitative analysis results, as shown in Figure 5H and Table S3, DLBCL patients (*n* = 2) demonstrated the highest overall tumor uptake (SUV_max_, 11.10 ± 4.79), followed by SCLC (SUV_max_, 7.51 ± 3.01, *n* = 30), NSCLC (SUV_max_, 3.20 ± 1.33, *n* = 4), and other types of tumors. The [^18^F]AlF-NOTA-QHY-04 uptake of NSCLC, TNBC, pancreatic cancer, glioma, HCC and CRC were all significantly lower than SCLC (*P* < 0.05), and showed no significant difference between each other (*P* > 0.05). Figure 5A and 5E show the images for 2 patients who underwent PET imaging with [^18^F]AlF-NOTA-QHY-04 and [^18^F]FDG respectively. Higher uptake of [^18^F]AlF-NOTA-QHY-04 than [^18^F]FDG was observed in the 10 lesions of the patient (No. 32) initially diagnosed with DLBCL (SUV_max_, 12.43 ± 4.85 vs. 8.00 ± 4.57, *P* = 0.062; SUV_mean_, 7.52 ± 3.01 vs. 4.39 ± 2.36, *P* = 0.025), especially in the inguinal lymph node (see blue arrow pointed in Figure 5A1) without discernible uptake of [^18^F]FDG but significant higher uptake of [^18^F]AlF-NOTA-QHY-04 (SUV_max_, 6.09 vs. 2.17). In contrast, [^18^F]FDG uptake was slightly higher than [^18^F]AlF-NOTA-QHY-04 uptake in the primary lesion (SUV_max_, 5.23 vs. 4.33), lymph node metastasis (SUV_max_, 4.26 vs. 2.75) and bone metastasis (SUV_max_, 4.1 vs. 3.18) of a NSCLC patient (No. 35, Figure 5E).

Furthermore, [^18^F]AlF-NOTA-QHY-04 PET/CT imaging findings were compared between primary tumors and metastases in 30 SCLC patients. There was no significant difference in radiotracer uptake between primary lesions, lymph node metastases, pleural metastases, hepatic metastases, bone metastases and adrenal metastases (*P* > 0.05), indicating uniform and high CXCR4 density on the primary and metastases in SCLC (Figure 5I). However, uptakes of these lesions were all significantly higher than that of brain metastases (*P* < 0.05), which may be explained by the hydrophilicity of the tracer making it difficult to cross the blood-brain barrier.

### PET imaging of brain tumors

**Figure 6:**
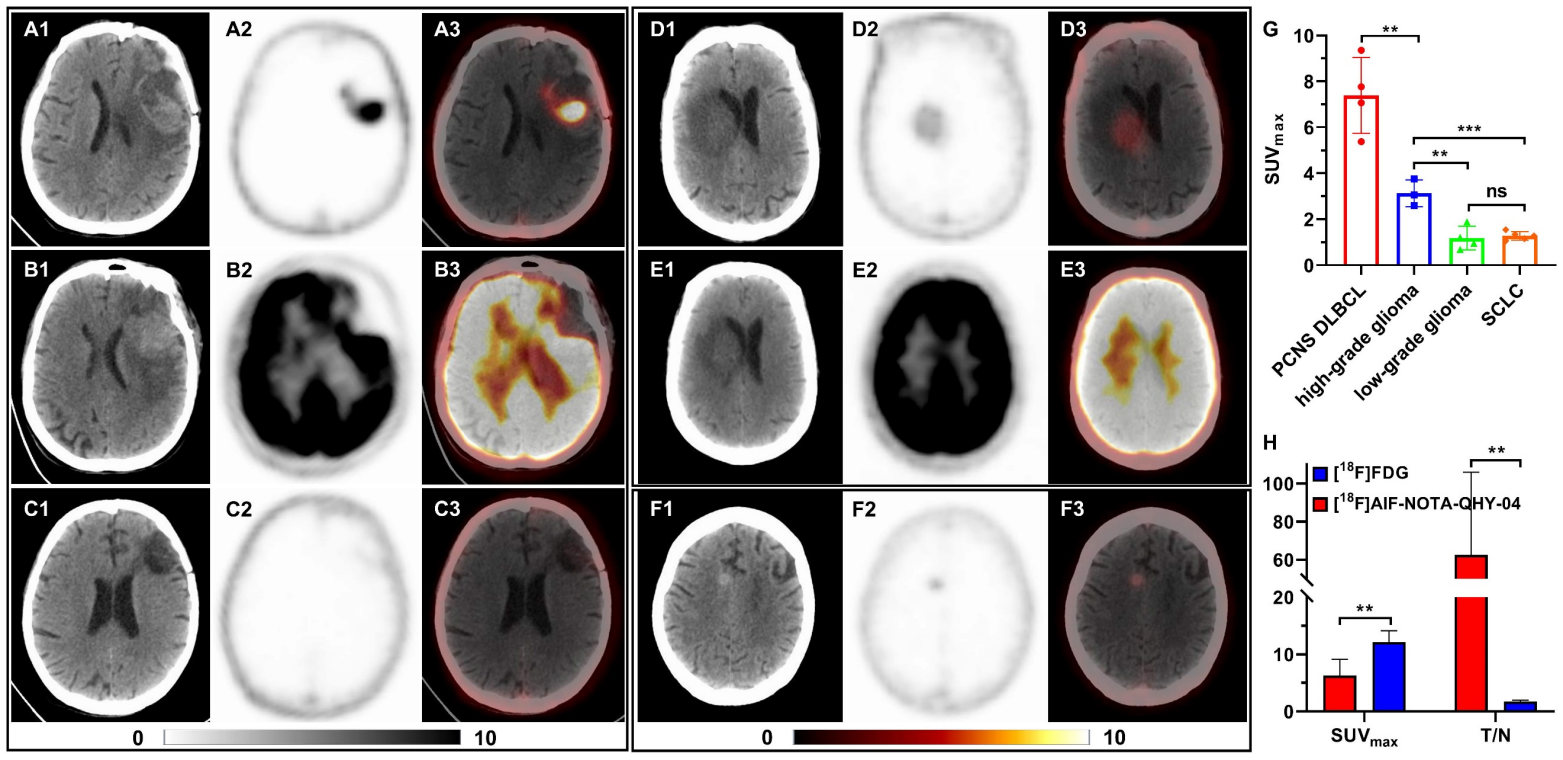
[^18^F]AlF-NOTA-QHY-04 PET/CT scanning of patients with brain tumors. (A-C) Representative PET images of a PCNS DLBCL patient, (A) [^18^F]AlF-NOTA-QHY-04 PET before treatment, (B) [^18^F]FDG PET before treatment, (C) [^18^F]AlF-NOTA-QHY-04 PET after complete response to therapy. Representative PET images of a low-grade glioma patient, (D) [^18^F]AlF-NOTA-QHY-04 PET, (E) [^18^F]FDG PET. (F) Representative PET images of a SCLC patient with brain metastasis. (G) SUV_max_ of brain tumors in 16 patients. (H) Bar chart displaying average T/N of PET imaging with the two tracers. ns, not statistically significant. **P < 0.01, ***P < 0.001.

16 patients with primary or metastatic brain tumors underwent [^18^F]AlF-NOTA-QHY-04 PET/CT imaging, including newly diagnosed or recurrent primary central nervous system diffuse large B-cell lymphoma (PCNS DLBCL, *n* = 4), high-grade gliomas (*n* = 3), low-grade gliomas (*n* = 4) and brain metastases of SCLC (*n* = 5). The representative images and relative uptake values are shown in Figure 6A-G. As depicted in Figure 6G, the average uptake of PCNS DLBCL was highest, followed by high-grade gliomas (7.39 ± 1.65 vs. 3.13 ± 0.58, *P* < 0.01). There was no significant difference between the low-grade gliomas and brain metastases of SCLC (1.18 ± 0.51 vs. 1.27 ± 0.18, *P* = 0.716), both significantly lower than high-grade gliomas (*P* < 0.01).

Moreover, three patients with PCNS DLBCL and one patient with low-grade glioma underwent PET imaging with two different tracers, [^18^F]FDG and [^18^F]AlF-NOTA-QHY-04. Figure 6A-C show the representative images of a PCNS DLBCL patient, who underwent PET imaging with the two tracers respectively before treatment and [^18^F]AlF-NOTA-QHY-04 PET imaging after therapy. Although the SUV_max_ of [^18^F]FDG was noticeably higher than that of [^18^F]AlF-NOTA-QHY-04, the tumor-to-normal brain ratio (T/N) of [^18^F]FDG was obviously lower than that of [^18^F]AlF-NOTA-QHY-04. More surprisingly, there was no uptake after a complete response to therapy, implying that [^18^F]AlF-NOTA-QHY-04 holds great potential for evaluating treatment efficacy accurately. Similar results were also observed in a low-grade glioma patient (Figure 6D and 6E). In addition, even though the uptake in brain metastases of SCLC was very low, the tumor lesion can still be clearly identified (Figure 6F). Collectively, the average SUV_max_ of [^18^F]FDG was significant higher than that of [^18^F]AlF-NOTA-QHY-04 (12.12 ± 2.03 vs. 6.29 ± 2.85, *P* = 0.011), but the average T/N of [^18^F]FDG was significant lower than that of [^18^F]AlF-NOTA-QHY-04 (1.70 ± 0.25 vs. 62.55 ± 43.24, *P* = 0.027) (Figure 6H). Hence, [^18^F]AlF-NOTA-QHY-04 PET may be feasible in brain tumor imaging and efficacy evaluation.

### mIF and IHC analysis

**Figure 7:**
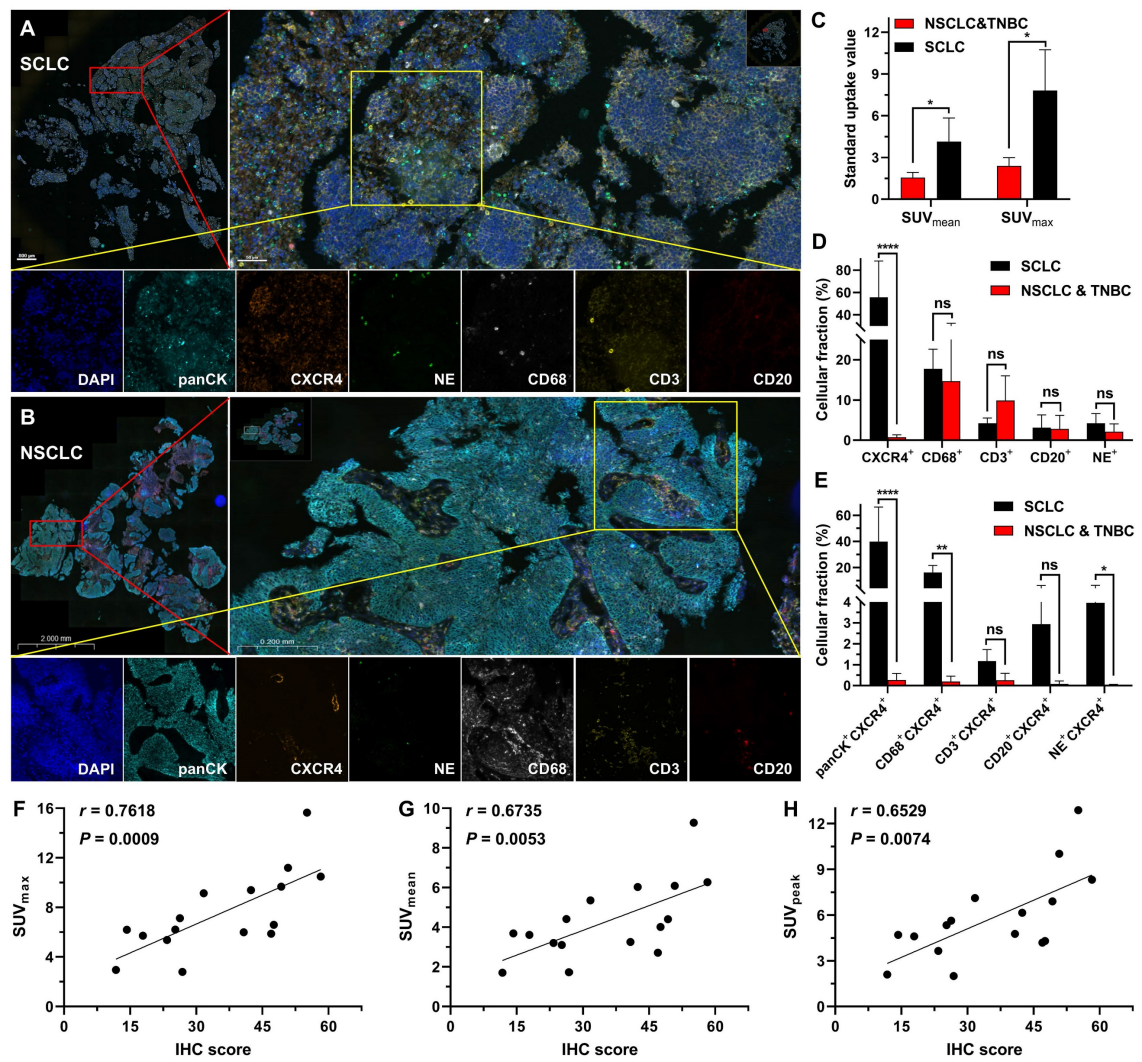
Histopathological characteristics of different tumor patients. Representative mIF images of tumor tissue samples obtained from (A) SCLC and (B) NSCLC patients. (C) SUV of [^18^F]AlF-NOTA-QHY-04 in patients. (D and E) mIF quantification of various cell density in SCLC (*n* = 3), NSCLC and TNBC (*n* = 3) tumor tissues. (F-H) Correlation between CXCR4-positive proportion of IHC (IHC score) and PET parameters. Spearman's rho (denoted as *r*) and *P* values are supplied in the graphs. ns, not statistically significant. *P < 0.05, **P < 0.01, ****P < 0.0001.

Through mIF staining of tumor tissues from the SCLC patients (the high uptake group) and NSCLC together with TNBC patients (the low uptake group), we observed that the high uptake group had a significantly higher level of CXCR4 expression than the low uptake group (NSCLC and TNBC) (Figure 7A-D), which corresponded to the positive correlation between IHC analysis of CXCR4 expression and tracer uptake in SCLC patients (Figure 7F-H). Additionally, we discovered that the high uptake of [^18^F]AlF-NOTA-QHY-04 in SCLC patients primarily depended on the high expression of CXCR4 in tumor cells, followed by macrophages and neutrophils in the tumor tissues (Figure 7E).

### Clinical impact of [^18^F]AlF-NOTA-QHY-04 PET/CT imaging

While not a primary endpoint of this study, [^18^F]AlF-NOTA-QHY-04 PET/CT imaging resulted in clinically significant findings in 6 patients (10.9% of the cohort). These included the detection of additional metastatic sites in 3 SCLC patients leading to upstaging, improved tumor delineation for radiotherapy planning in 2 PCNS DLBCL patients, and suggestion of higher-grade disease prompting repeat biopsy in 1 glioma patient.

## Discussion

CXCR4 has attracted considerable attention due to its intimate involvement in promoting the progression and metastasis of various tumors. With the emergence of novel CXCR4-targeted therapies and drugs, there is growing interest in developing CXCR4-targeted tracers 29. Among them, ^68^Ga-pentixafor represents a key development as it enables high-contrast PET imaging of CXCR4 expression *in vivo*, and is widely used in clinical trials 30,31.

Compared to ^68^Ga isotope, radiolabeling with ^18^F offers select advantages, such as the feasibility of producing large quantities and suitability for transport due to the longer half-life of ^18^F. Furthermore, the lower energy of positron particles emitted by ^18^F and the lower partial volume effect may lead to improved detection of smaller lesions 13. Hence, ^18^F-labeled CXCR4-targeting peptides show remarkable potential for molecular imaging characterization and monitoring of tumors. However, the high uptake of the most reported ^18^F-labeled tracers in the liver and other normal organs reduced the image contrast 15-18. The main objective of this study was to explore the potential application of the ^18^F-labeled CXCR4 tracer ([^18^F]AlF-NOTA-QHY-04) for cancer imaging. Successful radiosynthesis of [^18^F]AlF-NOTA-QHY-04 was achieved, with high radiochemical yield, radiochemical purity, and molar activity. Moreover, the specificity and capability of [^18^F]AlF-NOTA-QHY-04 for *in vivo* imaging of CXCR4 expression in tumor models and cancer patients were validated.

In xenograft models, [^18^F]AlF-NOTA-QHY-04 excreted through the kidneys due to its hydrophilicity and showed much lower non-specific accumulation compared to previously reported ^18^F-labeled CXCR4 tracers, such as Al[^18^F]NOTA-T140 15, [^18^F]AlF-NOTA-pentixather 16, and [^18^F]MCFB 17, which were associated with elevated digestive system uptake, especially in the gallbladder, liver, and intestine. The capability of [^18^F]AlF-NOTA-QHY-04 to quickly clear from nontarget tissues based on its optimized pharmacokinetic profile significantly contributed to the high-contrast PET images. Moreover, our studies indicated that [^18^F]AlF-NOTA-QHY-04 may offer advantages in tumor imaging by minimizing interference from background signals especially in the liver, lung, and spleen, despite exhibiting a modestly elevated renal uptake relative to ^68^Ga-Pentixafor 14,32.

In patient PET/CT studies, we found that DLBCL showed the highest uptake among 8 different kinds of cancers, while in solid tumors, uptake in the primary lesions of SCLC patients was significantly higher than the other solid tumors, as is consistent with a recent study investigating 690 patients imaged with ^68^Ga-Pentixafor 6. However, there was no significant difference in NSCLC, TNBC, pancreatic cancer, glioma, HCC and CRC. Furthermore, we elucidated for the first time that the high tracer uptake in certain solid patients is mainly due to the high expression of CXCR4 in tumor cells, followed by macrophages and neutrophils in the tumor tissues by mIF staining, which proved the PET signal provided by [^18^F]AlF-NOTA-QHY-04 indeed can accurately reflect the CXCR4 expression levels on multiple tumors.

CXCR4 is known as a biomarker for tumor malignancy in multiple types of cancer, and the higher its expression, the higher the degree of tumor malignancy, making it more prone to metastasis, drug resistance, and so on 1,4. As is well known, SCLC is a highly malignant tumor characterized by an exceptionally high proliferative rate, high metastatic rate and poor prognosis 33, which to some extent supports our clinical findings. According to reports, CXCR4 is highly expressed in B cells 34,35, while DLBCL is a highly malignant tumor originating from B lymphocytes, which may be the reason for the highest uptake of [^18^F]AlF-NOTA-QHY-04 in DLBCL patients. Moreover, PET imaging with dual tracers ([^18^F]AlF-NOTA-QHY-04 and [^18^F]FDG) in some patients showed that higher uptake of [^18^F]AlF-NOTA-QHY-04 was observed in certain lesions, particularly in lymphoma patients, implying the CXCR4-targeted PET is more suitable than [^18^F]FDG PET in the diagnosis of lymphoma. These results provide a theoretical basis for using [^18^F]AlF-NOTA-QHY-04 individually or together with [^18^F]FDG PET, offering crucial guidance for accurate cancer diagnosis and treatment, especially for lymphoma and SCLC.

Additionally, [^18^F]AlF-NOTA-QHY-04 exhibits good imaging efficacy in brain tumors, with higher uptake in high-grade gliomas compared to low-grade gliomas. The reason may be that the high-grade gliomas have a higher degree of malignancy, further confirming the above deduction. Another possible reason is the increased disruption of the blood-brain barrier in high-grade gliomas 36. Currently, magnetic resonance imaging is a critical diagnostic tool for brain tumors, but it is susceptible to movement artifacts and difficult to quantify diagnosis 37. Further, the most commonly used [^18^F]FDG PET has significant limitations in diagnosing and distinguishing brain tumors due to the relatively high uptake of glucose by the cerebral cortex 37, as shown in Figure 6B and 6E. However, [^18^F]AlF-NOTA-QHY-04 exhibits a significant higher T/N than [^18^F]FDG in brain tumor imaging. This advantage endows [^18^F]AlF-NOTA-QHY-04 PET/CT with the potential for more accurate and specific imaging of central nervous system lymphoma, and also provides support for the diagnosis, staging, and prognosis of gliomas.

The current study has certain limitations. The first is the small number of enrolled patients for other types of tumors except for SCLC. Particularly for HCC (*n* = 1) and CRC (*n* = 1), our findings should be considered preliminary at best. The results for these tumor types, while included for completeness, do not allow for robust conclusions about CXCR4 expression patterns or the utility of [^18^F]AlF-NOTA-QHY-04 PET/CT in these cancers. Secondly, we did not directly compare the difference of tumor uptake between [^18^F]AlF-NOTA-QHY-04 and ^68^Ga-pentixafor. Our findings underscore the need for larger, tumor-specific studies in the future. While our diverse cohort provided valuable initial insights, dedicated studies with larger sample sizes for each tumor type are necessary to validate our observations. Particularly for NSCLC, TNBC, pancreatic cancer, HCC and CRC, where our sample sizes were limited, future research should focus on recruiting larger cohorts to establish the full potential of [^18^F]AlF-NOTA-QHY-04 PET/CT in these malignancies.

## Conclusion

We developed an ^18^F-labeled CXCR4-targeting tracer with suitable characteristics, such as high *in vivo* stability and rapid clearance of activity, leading to a favorable tumor-to-background ratio. It is well-suited for imaging CXCR4 expression in hematological malignancies and SCLC, as well as for imaging and evaluating the treatment of primary brain tumors. Moreover, the high tracer uptake in certain patients is figured out to be due to the high expression of CXCR4 in tumor cells.

## Supplementary Material

Supplementary methods, figures and tables.

## Figures and Tables

**Table 1:** General Characteristics of Patients Enrolled in This Study.

Patient No.	Sex	Age (years)	ECOG score	Tumor type	TNM or grade	[^18^F]FDG
1	M	39	0	SCLC	cT4N3M1, IV	-
2	F	72	1	SCLC	cT3N2M1, IV	-
3	M	72	1	SCLC	cT2N3M1, IV	-
4	M	67	1	SCLC	cT4N3M1, IV	-
5	F	58	0	SCLC	cT2N3M1, IV	-
6	M	59	1	SCLC	cT2N3M1, IV	-
7	F	78	1	SCLC	cT4N3M1, IV	-
8	M	66	0	SCLC	cT3N2M1, IV	-
9	M	68	0	SCLC	cT1N3M1, IV	-
10	M	61	0	SCLC	cT1N3M1, IV	-
11	M	59	0	SCLC	cT2N3M0, III	-
12	M	78	0	SCLC	cT1N2M1, IV	-
13	F	80	0	SCLC	cT4N2M1, IV	-
14	M	68	0	SCLC	cT4N3M0, III	-
15	M	46	0	SCLC	cT4N3M1, IV	-
16	F	76	1	SCLC	cT2N0M0, I	-
17	M	54	0	SCLC	cT4N2M0, III	-
18	F	64	0	SCLC	cT1cN3M0, III	-
19	F	45	0	SCLC	cT4N3M1c, IV	-
20	M	43	0	SCLC	cT3N2M0, III	-
21	M	70	1	SCLC	cT2N3M0, III	-
22	F	74	0	SCLC	cT4N3M0, III	-
23	M	67	0	SCLC	cT3N2M1c, IV	-
24	F	69	0	SCLC	cT3N2M1c, IV	-
25	M	70	0	SCLC	cT2N3M0, III	-
26	M	69	0	SCLC	cT4N3M1, IV	-
27	F	71	1	SCLC	cT1bN1M0, II	-
28	M	71	0	SCLC	cT3N1M0, III	-
29	M	67	0	SCLC	cT2aN2M0, III	-
30	M	69	0	SCLC	cT4N2M0, III	-
31	M	58	0	DLBCL	II	+
32	M	54	0	DLBCL	IV	+
33	M	67	1	NSCLC, ADC	cT2N3M1, IV	-
34	F	69	0	NSCLC, ADC	cT2N3M0, III	-
35	F	56	0	NSCLC, ADC	cT1N1M1, IV	+
36	M	69	0	NSCLC, SCC	cT2N1M0, II	-
37	M	62	0	Pancreatic carcinoma	cT2N1M1, IV	-
38	M	56	1	Pancreatic carcinoma	cT3N1M1, IV	-
39	M	47	0	Pancreatic carcinoma	cT2N0M0, I	-
40	F	58	0	TNBC	pT1N0M0, Recurrence	-
41	F	51	0	TNBC	pT4N3M0, Recurrence	-
42	F	66	0	TNBC	pT4N0M0, Recurrence	-
43	M	71	1	CRC	cT3N+M1, IV	-
44	F	72	0	HCC	cT2N0M0, II	-
45	M	69	1	PCNS DLBCL	-	+
46	F	67	2	PCNS DLBCL	-	+
47	M	54	1	PCNS DLBCL	-	+
48	M	61	0	PCNS DLBCL	-	-
49	M	81	2	Glioma	WHO II	+
50	M	18	0	Glioma	WHO I	-
51	M	65	1	Glioma	WHO II	-
52	M	60	1	Glioma	WHO III	-
53	F	35	0	Glioma	WHO II	-
54	F	65	1	Glioma	WHO IV	-
55	F	59	1	Glioma	WHO IV	-

M, male; F, female; ADC, adenocarcinoma; SCC, squamous cell carcinoma.
