# Supplementary material for: PET imaging of CXCR4 expression using [18F]AlF-NOTA-QHY-04 for hematologic malignancy and solid tumors
Source: Theranostics. 2024 Sep 30;14(16):6337–49. doi: 10.7150/thno.99025 (PMC11488100; doi:10.7150/thno.99025)
Supplement: Supplementary file 1 — Supplementary methods, figures and tables. [file thnov14p6337s1.pdf]

Supplementary Material

Synthesis of $[^{18}F]$AlF-NOTA-QHY-04

Briefly, NOTA-QHY-04 was radiolabeled with $[^{18}F]F^-$, following procedures. 10-20 GBq $[^{18}F]F^-$ produced by medical cyclotron (MINITRACE Cyclotron, GE, USA) was captured with a QMA SepPak light cartridge (Waters Corporation, USA) and then eluted with 300-500 μL saline (0.9% NaCl). Then, the saline containing $[^{18}F]F^-$ was added to reaction vial, followed by 6-12 μL $AlCl_3$ (10 mM in 0.1M sodium acetate buffer, pH 4), 200 μL acetonitrile (≥ 99.9%, Sigma-Aldrich, USA), and 20-30 μL of NOTA-QHY-04 (20 mg/mL in metal-free water). The vial was heated for 10 min at 110°C. After cooling, the reaction system was diluted with 10 mL of water and was transferred to a Qasis light HLB cartridge (Waters Corporation, USA). The cartridge was then washed with 10 mL of water to remove the free $[^{18}F]F^-$. Finally, the desired product was eluted and collected with 0.6 mL of ethanol. The product was reconstituted in saline and passed through a 0.22 μm syringe filter (Pall Corporation, USA) into a sterile vial.

Octanol-water partition coefficient (logD)

The partition coefficient (logD) of $[^{18}F]$AlF-NOTA-QHY-04 was determined in a phosphate-buffered saline (PBS, 0.1 M, pH 7.4) and 1-octanol mixture (1:1 v/v) as the following method. Initially, a 10 mL centrifuge tube was loaded with 0.1 mL of $[^{18}F]$AlF-NOTA-QHY-04 (37-74 KBq), followed by the addition of 1.9 mL of PBS and 2.0 mL of 1-octanol. The mixture was vortexed for 1 minute to ensure thorough mixing and then centrifuged at 5000 rpm for 3 minutes to separate the phases. Three 100 μL samples from each phase were measured for radioactivity using a γ-counter. This procedure was repeated three times to ensure the accuracy of the results. The logD was calculated by dividing the average radioactivity in the 1-octanol layer by that in the PBS layer, and the value was reported as mean ± SD.

In vitro and *in vivo* stability

The *in vitro* stability of $[^{18}F]$AlF-NOTA-QHY-04 in physiological saline and 5%

human serum albumin solution were evaluated by radio-HPLC to examine the radiochemical purity after different incubation periods. For the saline study, 200 μL of [^{18}F]AlF-NOTA-QHY-04 (0.74–3.70 MBq) was incubated at 37°C with 800 μL of saline for 1 h, 3 h and 6 h respectively, and then directly evaluated by radio-HPLC. For the 5% human serum albumin solution study, 200 μL of [^{18}F]AlF-NOTA-QHY-04 (1.85–3.70 MBq) was added to 800 μL of 5% human serum albumin solution incubated at 37 °C with slight agitation for 1 h, 3 h and 6 h, then the samples were precipitated by additional acetonitrile (300 μl) and removed by centrifugation at 13000 rpm for 5 min. Finally, the supernatant was collected and passed through a 0.22μm membrane filter (Millipore, USA) and analyzed by radio-HPLC.

The metabolic stability *in vivo* was performed on BALB/c nude mice (female, 5-6 weeks old). Briefly, BALB/c nude mice were intravenously injected with 37 MBq of [^{18}F]AlF-NOTA-QHY-04. The mice were sacrificed at 30 min and 60 min post injection (p.i.), and the blood and urine were collected. The blood samples were centrifuged at 13,000 rpm for 5 min, then the blood supernatants were precipitated by additional acetonitrile and were removed by centrifugation. The urine sample was directly diluted with 1 mL of PBS. The processed blood supernatants and diluted urine samples were collected and passed through a 0.22 μm membrane filter (Millipore, USA) and analyzed by radio-HPLC.

Biolayer interferometry (BLI) binding assays

To assess the binding characteristics of the unlabeled precursor (NOTA-QHY-04) to CXCR4, the biolayer interferometry Octet Red96 system (PALL ForteBio, USA) was employed. Initially, the recombinant CXCR4 protein (Novoprotein, Cat. No.: C16M, MW: 30.6 KD) was biotinylated using the EZ-link Sulfo-NHS-LC-biotinylation kit (Thermo Scientific, cat. 21435). The protein mixture of CXCR4 and biotin, in a 1:1 molar ratio, was prepared in HEPES buffered saline (HBS) buffer (10 mM HEPES, 150 mM NaCl, pH 7.4) and incubated on ice for 2 h. Excess biotin was removed by dialyzing the reaction mixture in HBS buffer. All assays were performed at 30 °C using HBS-P buffer (10 mM HEPES pH

7.4, 150 mM NaCl, 0.005% tween-20) with continuous shaking at 1000 rpm. Biotinylated CXCR4 was immobilized on Super Streptavidin (SSA) biosensors by immersing the sensors in a protein solution of 35 μg/mL. Inactive reference controls were established by immobilizing biotin-labeled streptavidin protein on SSA sensors. To determine the binding affinity, a flowing phase with six gradient concentrations (0.0313, 0.0625, 0.125, 0.25, 0.5, and 1 μM NOTA-QHY-04 sample) was prepared and flowed. The association and dissociation periods were both set to 120 seconds. Prior to fitting, the data were baseline-subtracted and analyzed using a 1:1 binding model and the ForteBio data analysis software. Mean K_d value was determined by applying a global fit to all the data.

Cell culture and tumor-bearing models

Six human tumor cell lines (A549, Daudi, NCI-H69, U251, MDA-MB-231 and MIA PaCa-2) obtained from the FuHeng Biology (China) were used in this study. The A549/CXCR4 cell line, which stably expressed human CXCR4, was generated by lentivirus (Syngenbio, China) transfection and then selected using 2 μg/mL of puromycin (Beyotime) for 2 weeks. Daudi and NCI-H69 were cultured in RPMI-1640 medium (Gibco), the other cells were maintained in DMEM medium (Gibco) supplemented with 10% FBS (ExCell Bio, China) plus 1% penicillin and streptomycin (Solarbio, China) at 37 ^oC in a humidified incubator containing 5% CO_2.

The experiments involving animals were performed in accordance with the guidelines of the Animal Care and Use Committee of Shandong Cancer Hospital and Institute, the Shandong First Medical University (Jinan, China). BALB/c nude mice and SCID mice (female, 5-6 weeks old) were obtained from the Beijing HFK Bioscience Co., Ltd. (Beijing, China). Approximately 6×10^6 Daudi cells and the other human tumor cells suspended in 100 μL of PBS were implanted subcutaneously into the right flank of SCID mice and BALB/c nude mice, respectively. The mice were used for *in vivo* studies when the tumor volume reached the size of 200–500 mm^3.

Flow cytometry and immunofluorescence cell staining

Cells (1×10^6) were collected and washed with PBS twice, stained with PE-conjugated anti-human CD184 (CXCR4) recombinant antibody (304504, BioLegend) and the PE-conjugated Mouse IgG1 isotype control antibody (400111, BioLegend) for 40 min at room temperature. After washing, the cells were analyzed by the BD LSRFortessa flow cytometer (Becton Dickinson, USA).

For immunofluorescence staining, A549, A549/CXCR4, U251, MDA-MB-231 and MIA PaCa-2 cells (8×10^4 cells per dish) were seeded in 35-mm glass bottom cell culture dishes and incubated overnight. NCI-H69 cells were immobilized onto the Shi-fix coverslips (Target Technology) according to the manufacturer's instructions. After rinsing with PBS once, cells were fixed with 4% paraformaldehyde for 20 min at room temperature. Cells were then washed with PBS (3 × 5 min) and blocked for 10 min at room temperature with blocking buffer (Beyotime). Subsequently, cells were incubated with anti-CXCR4 rabbit monoclonal antibody (1:500, ab124824, Abcam) in primary antibody dilution buffer (Beyotime) overnight at 4 ^{o}C. Then the cells were washed with immunostaining detergent (Beyotime), 3 × 5 min, and incubated with Alexa Fluor 488 goat anti-rabbit IgG secondary antibody (1:500, ab150081, Abcam) in secondary antibody dilution buffer (Beyotime) for 1 h at room temperature protected from light. After washing with immunostaining detergent for three times, the cells were incubated with DAPI solution (Solarbio) for 10 min at room temperature. Following another washing step, the cells were mounted with antifading mounting medium (Solarbio) and stored at -20 ^{o}C before imaging. Finally, the cells were imaged by ZEISS LSM 800 confocal laser scanning microscope using 63× oil immersion objective.

For cell uptake study, A549 and A549/CXCR4 cells (4×10^5) were seeded in 6-well plates and grown to 90% confluence. After washing with PBS, the cells were incubated with [^{18}F]AlF-NOTA-QHY-04 (37 kBq/well), with or without NOTA-QHY-04 precursor (2 μg/well) as blocking agent at 37 ^{o}C for 30, 60, and 90 min. Then cells were washed three times with cold PBS (1 mL) to eliminate unbound radiotracer, and lysed in 1 mL of 1.0 M

NaOH. Finally, the cell lysates were collected and measured by an automatic gamma counter (2470 Wizard2, PerkinElmer). Cellular uptake of [^{18}F]AlF-NOTA-QHY-04 was indicated as the percentage of added radioactive dose.

Small-animal PET imaging

The tumor-bearing mice were injected with 3.70 ± 0.74 MBq of [^{18}F]AlF-NOTA-QHY-04 in about 150 μL saline via the tail vein. All the PET scans were performed with micro-PET/CT (IRIS PET/CT, Inviscan, France). Static PET images were acquired at 60 min after radiotracer injection. Dynamic PET scans were acquired within 60 min immediately after administration. After dynamic scanning for 1 h and 3 h, all the mice underwent two static scans respectively to obtain 2-h and 4-h PET imaging data after injection. For the blocking study, excess dose (5 mg/kg body weight) of unlabeled precursor was intraperitoneally injected 1 h prior to [^{18}F]AlF-NOTA-QHY-04 injection. All the mice were anesthetized with 2.5% isoflurane in an air mixture. The PET/CT images were analyzed using OsiriX MD software (Pixmeo SARL, Geneva, Switzerland), and the regions of interest (ROIs) were drawn along the boundary covering the whole tumor volume for quantification of the radioactivity (expressed as %ID/mL).

PET/CT imaging in cancer patients

A total of 55 patients including 8 tumor types were recruited for PET/CT imaging. Histological confirmation was obtained for all patients prior to enrollment, following current clinical practice guidelines for each type of cancer. For solid tumors, image-guided core needle biopsies of primary tumors or accessible metastatic sites were performed. While some cases, such as HCC, were diagnosed based on characteristic imaging findings. Lymphoma cases underwent excisional biopsies of lymph nodes or stereotactic biopsies of brain lesions for DLBCL. Glioma cases had stereotactic or open surgical biopsies as appropriate. For metastatic lesions, especially in SCLC, diagnosis often relied on a combination of primary tumor histology, imaging characteristics, and multidisciplinary clinical assessment. All specimens were evaluated by experienced

pathologists following standard diagnostic criteria, including routine H&E staining and relevant immunohistochemical analyses. Histological confirmation was completed before patients underwent [^{18}F]AlF-NOTA-QHY-04 PET/CT imaging to ensure an accurate correlation between imaging findings and tumor characteristics.

All the PET/CT scans in patients were acquired through an integrated in-line PET/CT system (GEMINI TF Big Bore, Philips Healthcare, USA) at 60 min after intravenous administration of 4.81 MBq/kg [^{18}F]AlF-NOTA-QHY-04 or [^{18}F]FDG. The attenuation-corrected fused PET/CT images were displayed and analyzed using a nuclear medical information system (Beijing Mozi Healthcare Technology Co., Ltd., Beijing, China). ROIs were drawn on the transaxial images, and the maximum, mean, and peak standard uptake value (SUV_{max}, SUV_{mean}, SUV_{peak}) were calculated automatically and used for measuring the uptake of [^{18}F]AlF-NOTA-QHY-04 by malignant tissues. In PET imaging of brain tumors, T/N (tumor-to-normal brain ratio) was calculated according to the ratio of SUV_{max} of tumor to SUV_{mean} of the contralateral normal brain tissue. Two experienced nuclear medicine physicians unaware of the patients' clinical history analyzed the PET/CT data first independently and then in consensus.

Immunohistochemical and multicolor immunofluorescence (mIF) staining studies

For xenograft models, the animals were euthanized after *in vivo* scanning, tumor tissues were then harvested and fixed with formalin and embedded with paraffin. The formalin-fixed paraffin-embedded (FFPE) tumor tissues from xenograft models and biopsy or surgical specimens of patients were cut into 4-μm-thick sections used for IHC and multicolor immunofluorescence (mIF) staining. The anti-CXCR4 rabbit monoclonal antibody (1:200, ab124824, Abcam) was used for assessment of CXCR4 expression. IHC slides were scanned using a ZEISS Automatic Digital Slide Scanner Axio Scan.Z1.

For mIF staining, the FFPE sections were baked for 2 h at 60 °C and then deparaffinized with xylene and rehydrated through decreasing gradient ethanol. Antigen retrieval was performed in AR6 buffer (Akoya Biosciences) in a microwave oven for 15

min. Endogenous peroxidase was deactivated by incubation with 3% H_2O_2 for 10 min to prevent false-positive staining. Multiplex immunofluorescence was performed by six rounds of staining, each including a protein block with 1% BSA followed by primary antibody and corresponding secondary horseradish peroxidase-conjugated antibody against mouse or rabbit immunoglobulins (Akoya Biosciences). Then the slides were incubated with different Opal fluorophore (1:100) diluted in 1X Plus Amplification Diluent (Akoya Biosciences) for 10 min in the dark. After tyramide signal amplification (TSA) and covalent linkage of the individual Opal fluorophores (Akoya Biosciences) to the relevant epitopes, the primary and secondary antibodies were removed via antigen retrieval as previously mentioned and the next cycle of immunostaining was initiated until the samples were labeled with all six markers. The sequence of primary antibodies and Opal fluorophores for biopsy or surgical specimens of patients was anti-panCK/Opal 480, anti-CD3/Opal 570, anti-NE/Opal 520, anti-CXCR4/Opal 620, anti-CD20/Opal 690, and anti-CD68/Opal 780. Finally, all the slides were counterstained with spectral DAPI (Akoya Biosciences) for 10 min and mounted with anti-fade fluorescence mounting medium (ab104135, Abcam) and stored at 4 ^{o}C before imaging. The multiplex immunofluorescence slides were scanned with the Akoya Vectra Polaris.

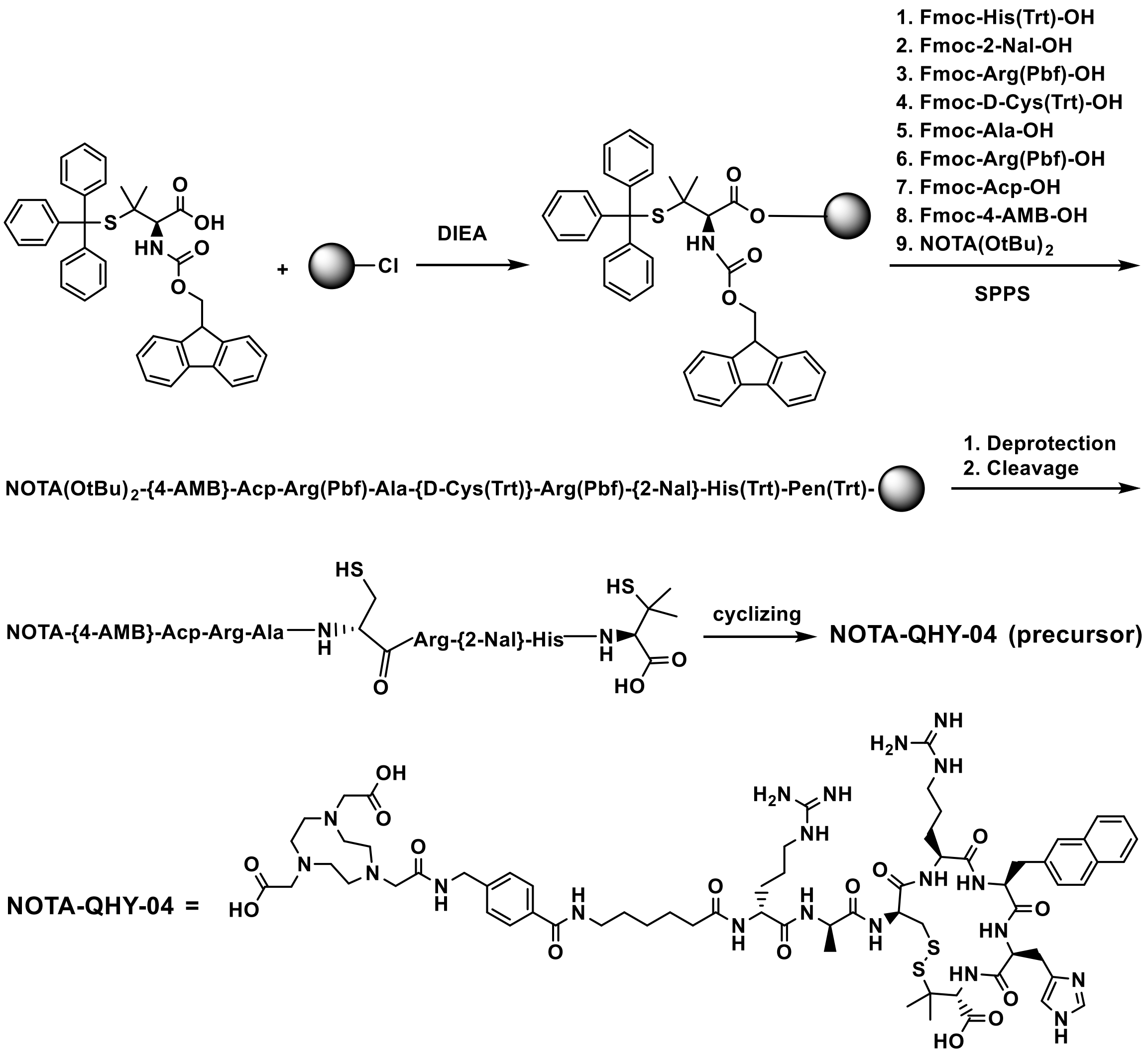

Figure S1. The synthetic routes of the precursor NOTA-QHY-04.

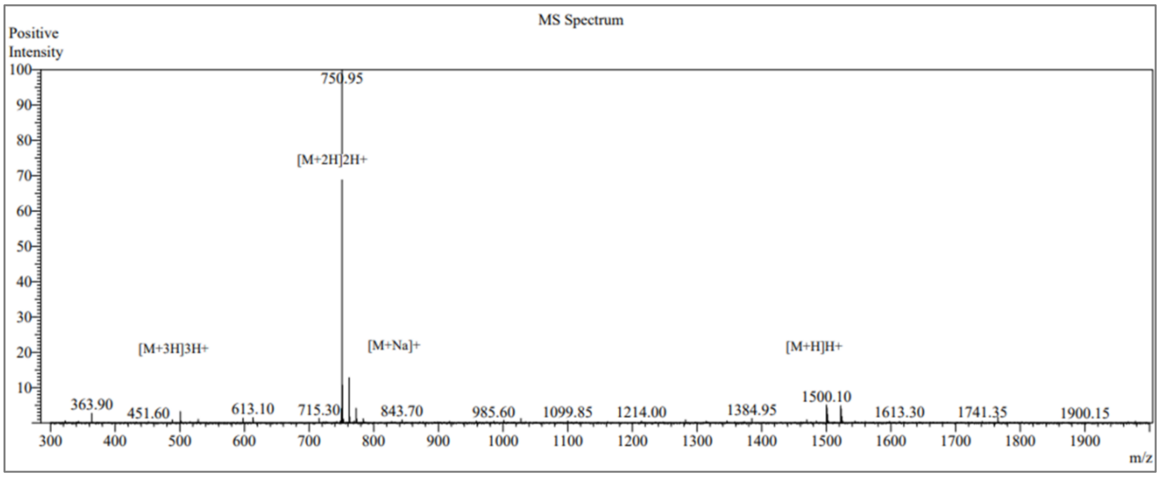

Figure S2. Mass spectra of NOTA-QHY-04. MS (ESI): *m*/*z* calculated for $C_{68}H_{99}N_{20}O_{15}S_2$: 1499.84, found: *m*/*z* = 1500.10 $[M+H]^+$.

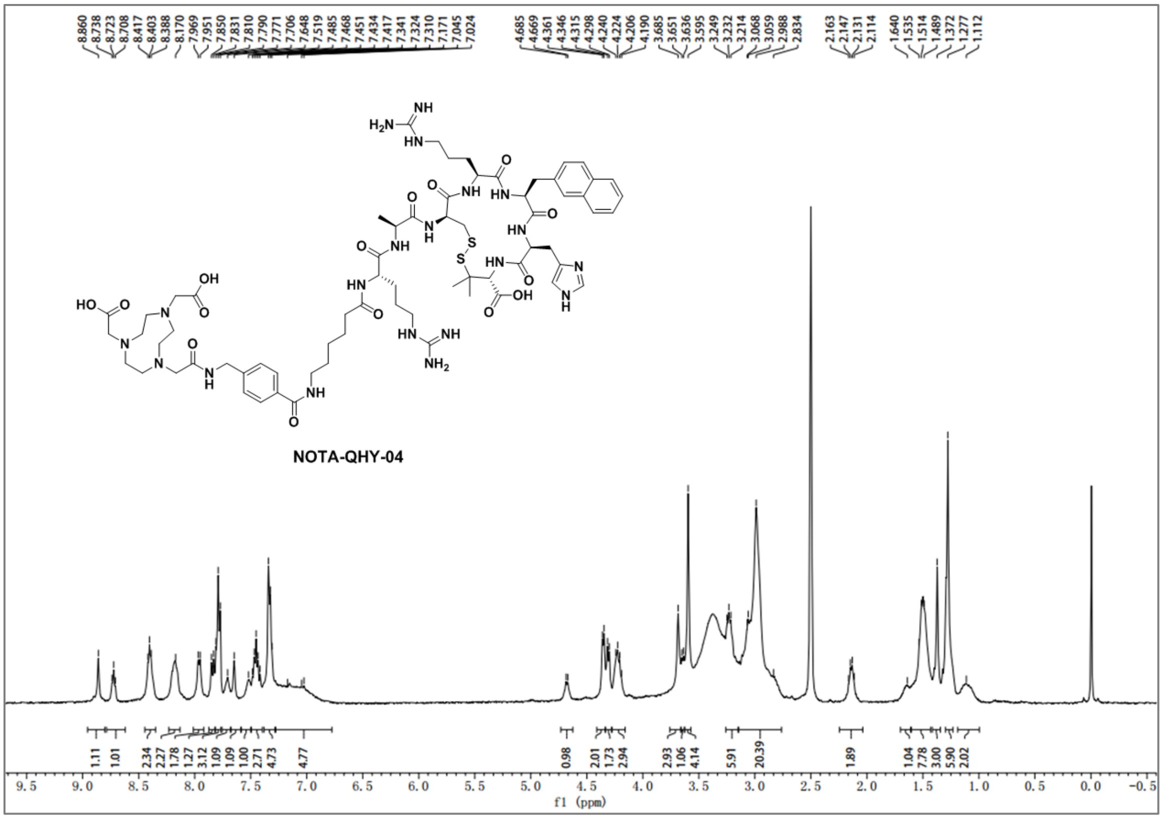

Figure S3. ^{1}H NMR (400 MHz, DMSO-d_6) of NOTA-QHY-04. ^{1}H NMR (400 MHz, DMSO-d_6, 25 ºC, δ): 8.86 (s, 1H), 8.72 (t, J = 6.0 Hz, 1H), 8.40 (t, J = 5.6 Hz, 2H), 8.17 (s, 2H), 7.96 (d, J = 7.2 Hz, 2H), 7.84 (d, J = 7.6 Hz, 1H), 7.79 (t, J = 8.0 Hz, 3H), 7.71 (s, 1H), 7.65 (s, 1H), 7.52 (s, 1H), 7.49–7.40 (m, 3H), 7.38–7.28 (m, 5H), 7.28–6.78 (m, 5H), 4.68 (d, J = 6.4 Hz, 1H), 4.35 (d, J = 6.0 Hz, 2H), 4.31 (d, J = 6.8 Hz, 2H), 4.28–4.16 (m, 3H), 3.68 (s, 3H), 3.64 (d, J = 6.0 Hz, 1H), 3.60 (s, 4H), 3.23 (t, J = 6.8 Hz, 6H), 3.14–2.76 (m, 20H), 2.14 (q, J = 6.4 Hz, 2H), 1.64 (s, 1H), 1.60–1.43 (m, 8H), 1.37 (s, 3H), 1.28 (s, 6H), 1.11 (s, 2H).

Table S1. Test items, acceptance criteria and test results for [^{18}F]AlF-NOTA-QHY-04.

Test item	Criteria	Test result
Appearance	Colorless and particle-free	Colorless
pH	5.0-8.0	6.0-7.0
Radiochemical purity	≥ 90%	≥ 98%
Residual ethanol	≤ 10% v/v	≤ 5% v/v
Residual acetonitrile	≤ 0.1% v/v	≤ 0.1% v/v
Radionuclide identity-approximate half-life ($t_{1/2}$)	$t_{1/2}$ = 110 ± 5 min	$t_{1/2}$ = 110 ± 5 min
Total radioactivity	50-5000 MBq/mL	652 ± 44 MBq/mL
Molar activity	≥ 3 GBq/μmol	30 ± 3 GBq/μmol
Sterility	No growth after 14 days of incubation at 37 °C	-
Bacterial endotoxins	≤ 15 EU per mL	0.2-0.5 EU per mL

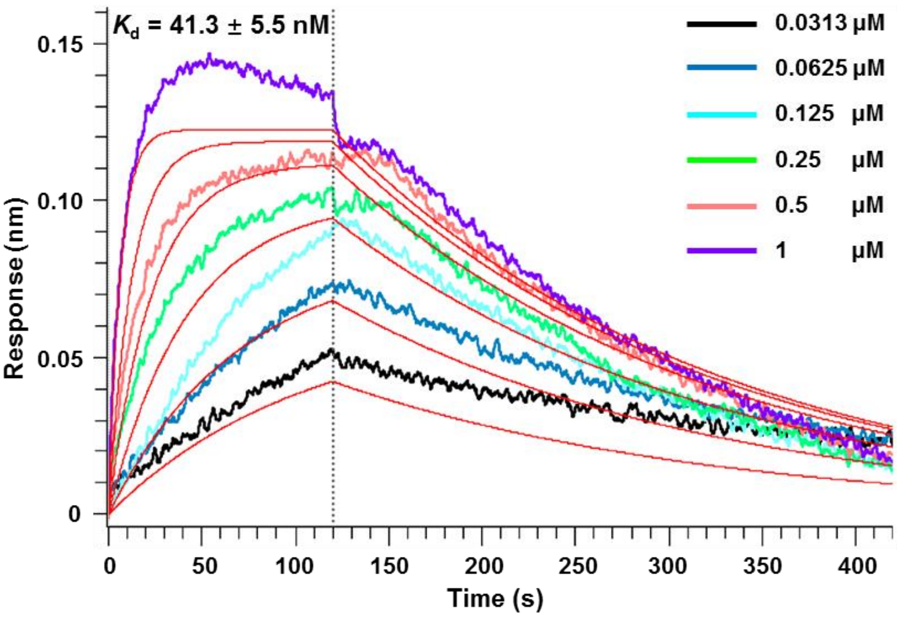

Figure S4. Determination of the binding affinity between CXCR4 and [natF]AlF-NOTA-QHY-04 by biolayer interferometry.

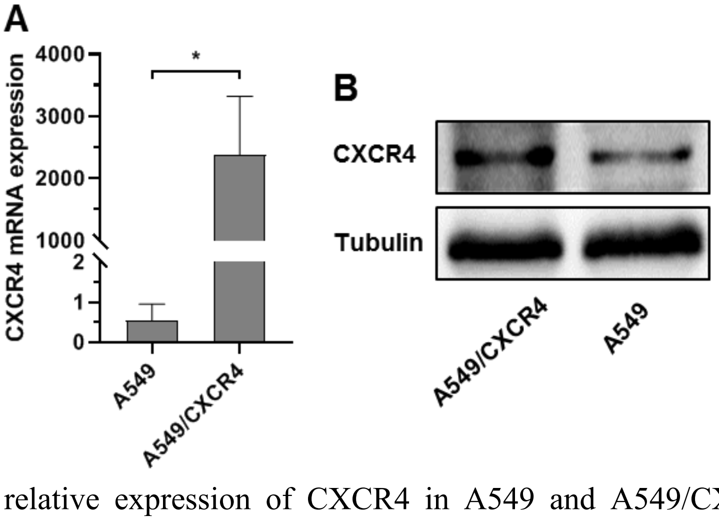

Figure S5. The relative expression of CXCR4 in A549 and A549/CXCR4 cell lines according to qRT-PCR (A) and WB (B). *$P < 0.05$.

Table S2. *Ex vivo* biodistribution data of [^{18}F]AlF-NOTA-QHY-04 in A549/CXCR4 tumor-bearing mice.

Organ	Tracer	
	[^{18}F]AlF-NOTA-QHY-04 (*n* = 3)	[^{18}F]AlF-NOTA-QHY-04 + NOTA-QHY-O4 (*n* = 3)
Blood	0.27 ± 0.05	0.29 ± 0.06
Brain	0.02 ± 0.01	0.02 ± 0.00
Heart	0.12 ± 0.02	0.11 ± 0.02
Liver	0.52 ± 0.04	0.33 ± 0.02
Spleen	0.33 ± 0.09	0.22 ± 0.02
Lung	0.42 ± 0.04	0.30 ± 0.03
Kidney	4.20 ± 1.09	2.81 ± 0.35
Stomach	0.13 ± 0.08	0.05 ± 0.00
Intestines	0.15 ± 0.07	0.13 ± 0.06
Muscle	0.10 ± 0.06	0.11 ± 0.07
Bone	0.25 ± 0.10	0.08 ± 0.03
tumor	0.95 ± 0.09	0.62 ± 0.12
Tumor-to-organ ratio		
T/Blood	3.63 ± 0.56	
T/Heart	7.77 ± 0.60	
T/Liver	1.84 ± 0.17	
T/Kidney	0.23 ± 0.05	
T/Muscle	10.94 ± 5.33	

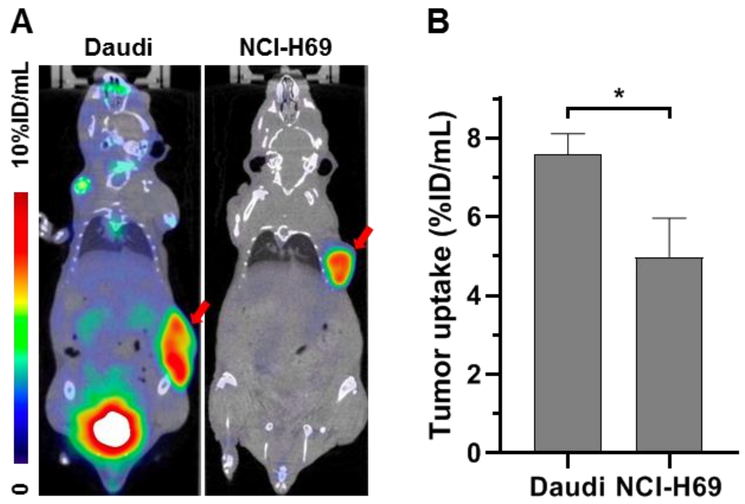

Figure S6. Representative static PET images (A) and quantification of [^{18}F]AlF-NOTA-QHY-04 uptake (B) in Daudi and NCI-H69 tumor-bearing mice ($n \geq 3$). *$P < 0.05$.

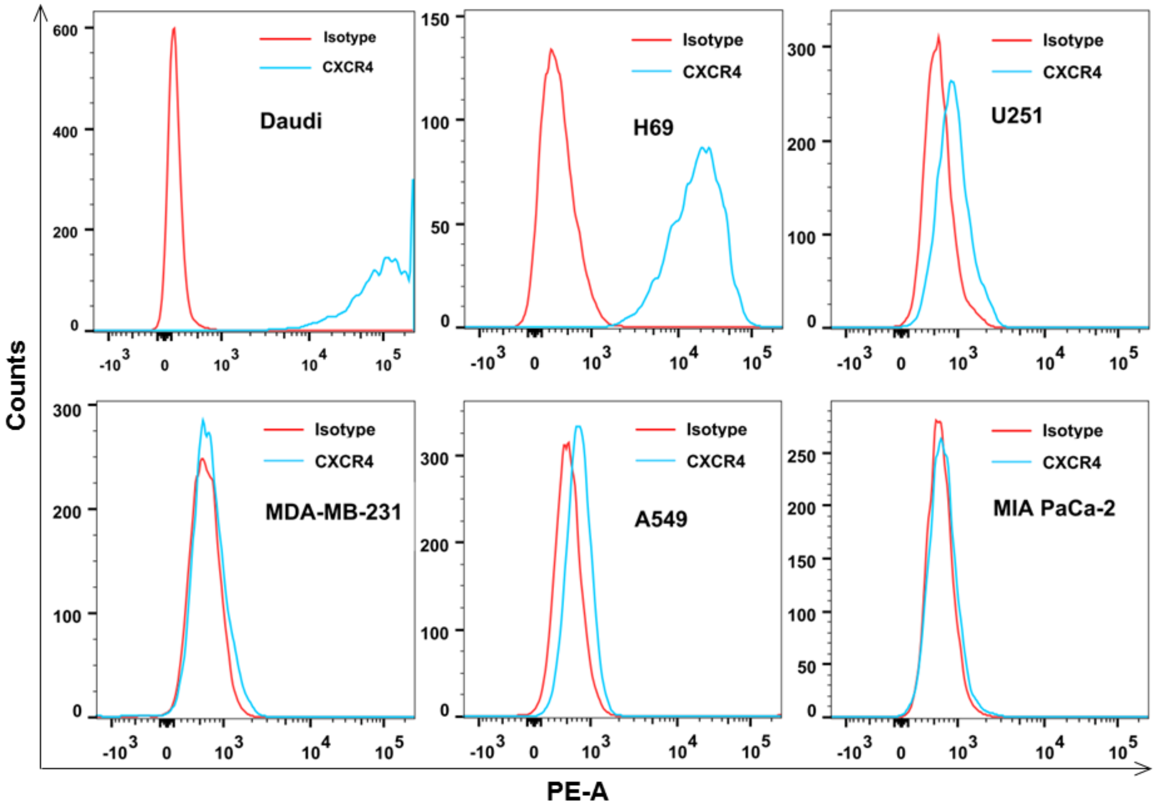

Figure S7. Flow cytometry histograms of different tumor cell lines incubated with PE-conjugated anti-human CD184 (CXCR4) antibody along with the isotype control antibody.

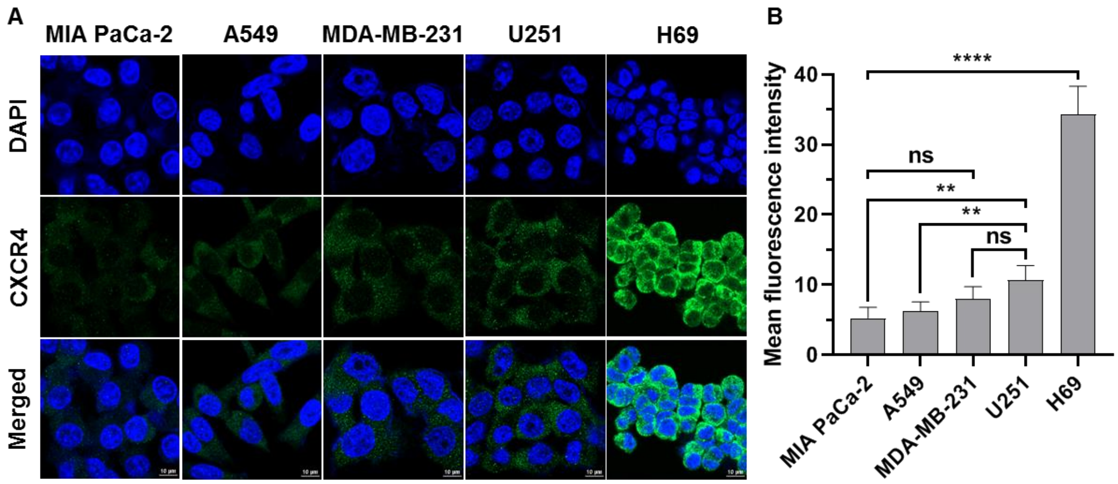

Figure S8. (A) Representative IF staining of MIA PaCa-2, A549, MDA-MB-231, U251 and H69 cells with the anti-CXCR4 antibody and DAPI. Scale bar = 10 μm. (B) The relative expression of CXCR4 in different tumor cell lines determined by IF staining. The mean fluorescence intensity was analyzed by ZEN 2012 (blue edition) software (Carl Zeiss).

Table S3. Standard uptake values of [^{18}F]AlF-NOTA-QHY-04 in different types of tumor patients.

Tumor type	Number of patients	SUV_{max}	SUV_{mean}
DLBCL	2	11.10 ± 4.79	6.78 ± 2.90
SCLC	30	7.51 ± 3.01	4.38 ± 1.85
NSCLC	4	3.20 ± 1.33	2.08 ± 0.79
TNBC	3	3.76 ± 1.51	2.13 ± 0.83
Pancreatic cancer	3	2.04 ± 0.80	1.24 ± 0.04
Glioma	7	2.02 ± 1.15	1.16 ± 0.73
Hepatocellular carcinoma	1	3.64	2.11
Colorectal cancer	1	2.29	1.42

DLBCL = diffuse large B-cell lymphoma; SCLC = small cell lung cancer; NSCLC = non-small cell lung cancer; TNBC = triple negative breast cancer.
